# Spontaneous Uterine Artery Rupture in Pregnancy, With Subsequent Severe Foetal Intracranial Haemorrhage

**DOI:** 10.7759/cureus.108228

**Published:** 2026-05-04

**Authors:** Omar Lotfi, Ahmed Ellaboudy, Supreet Mahurkar, Soneya Chowdhury, Reem Elnemr

**Affiliations:** 1 Obstetrics and Gynaecology, The Grange University Hospital, Cwmbran, GBR; 2 Obstetrics and Gynaecology, Zagazig University Hospitals, Zagazig, EGY

**Keywords:** foetal intracranial haemorrhage, hemoperitoneum in pregnancy, laparoscopic management, maternal hypovolemic shock, pregnancy complications, spontaneous uterine artery rupture, tetra-ventriculomegaly, uteroplacental insufficiency

## Abstract

Spontaneous rupture of the uterine artery during pregnancy is an exceptionally rare and life-threatening obstetric emergency. It often presents with sudden hemodynamic instability in the absence of external bleeding, posing diagnostic and management challenges. While maternal outcomes depend on timely intervention, foetal outcomes may be significantly affected by acute uteroplacental insufficiency.

We report a case of a 23-year-old gravida 2 para 1+0 woman at 26 weeks of gestation who presented with sudden hypovolemic shock without external haemorrhage. A prior anomaly scan at 23 weeks was unremarkable. Emergency laparoscopic exploration revealed approximately 3L of hemoperitoneum due to a ruptured branch of the left uterine artery, which was successfully ligated. The patient received eight units of blood transfusion and required intensive care support for 5 days.

Subsequent foetal follow-up revealed severe neurological sequelae. Ultrasound at 32 weeks demonstrated marked tetra-ventriculomegaly with features suggestive of intracranial haemorrhage. Repeat imaging at 35 weeks confirmed persistence of findings along with foetal growth restriction. The patient delivered at 38 weeks, and postnatal imaging confirmed hydrocephalus requiring ventriculoperitoneal shunt placement.

This case highlights the rare occurrence of spontaneous uterine artery rupture and its significant foetal neurological consequences, emphasizing the importance of early recognition and vigilant foetal surveillance following maternal hypovolemic events.

## Introduction

Spontaneous intra-abdominal haemorrhage during pregnancy is a rare but potentially life-threatening condition that poses significant diagnostic and therapeutic challenges. Among its various aetiologies, rupture of uterine or utero-ovarian vessels represents an uncommon cause, particularly in the absence of identifiable risk factors such as trauma, endometriosis, prior pelvic surgery, or underlying vascular abnormalities [1,2,3,4].

The clinical presentation is often subtle and nonspecific, typically manifesting as sudden abdominal pain accompanied by signs of hypovolemic shock without overt vaginal bleeding. This atypical presentation can delay diagnosis and intervention, increasing the risk of adverse maternal outcomes [2]. Early recognition and prompt surgical management remain crucial for maternal survival [3].

Foetal outcome in such cases is largely determined by the severity and duration of maternal hemodynamic compromise. Acute maternal hypovolemia may result in transient uteroplacental insufficiency, leading to foetal hypoxia-ischemia. This, in turn, can precipitate significant neurological injury, including intracranial haemorrhage and progressive ventriculomegaly [5,6].

In this report, we describe a rare case of spontaneous rupture of a uterine arterial branch during the second trimester, presenting with massive hemoperitoneum and maternal shock, followed by delayed but severe foetal intracranial pathology.

## Case presentation

A 23-year-old woman, gravida 2 para 1+0, with a history of one previous caesarean section, presented at 26 weeks of gestation with a sudden onset of symptoms suggestive of hypovolemic shock. Her medical and surgical history was otherwise unremarkable, with no known coagulopathy, no prior episodes of spontaneous bleeding, and no relevant family history.

**Figure 1:**
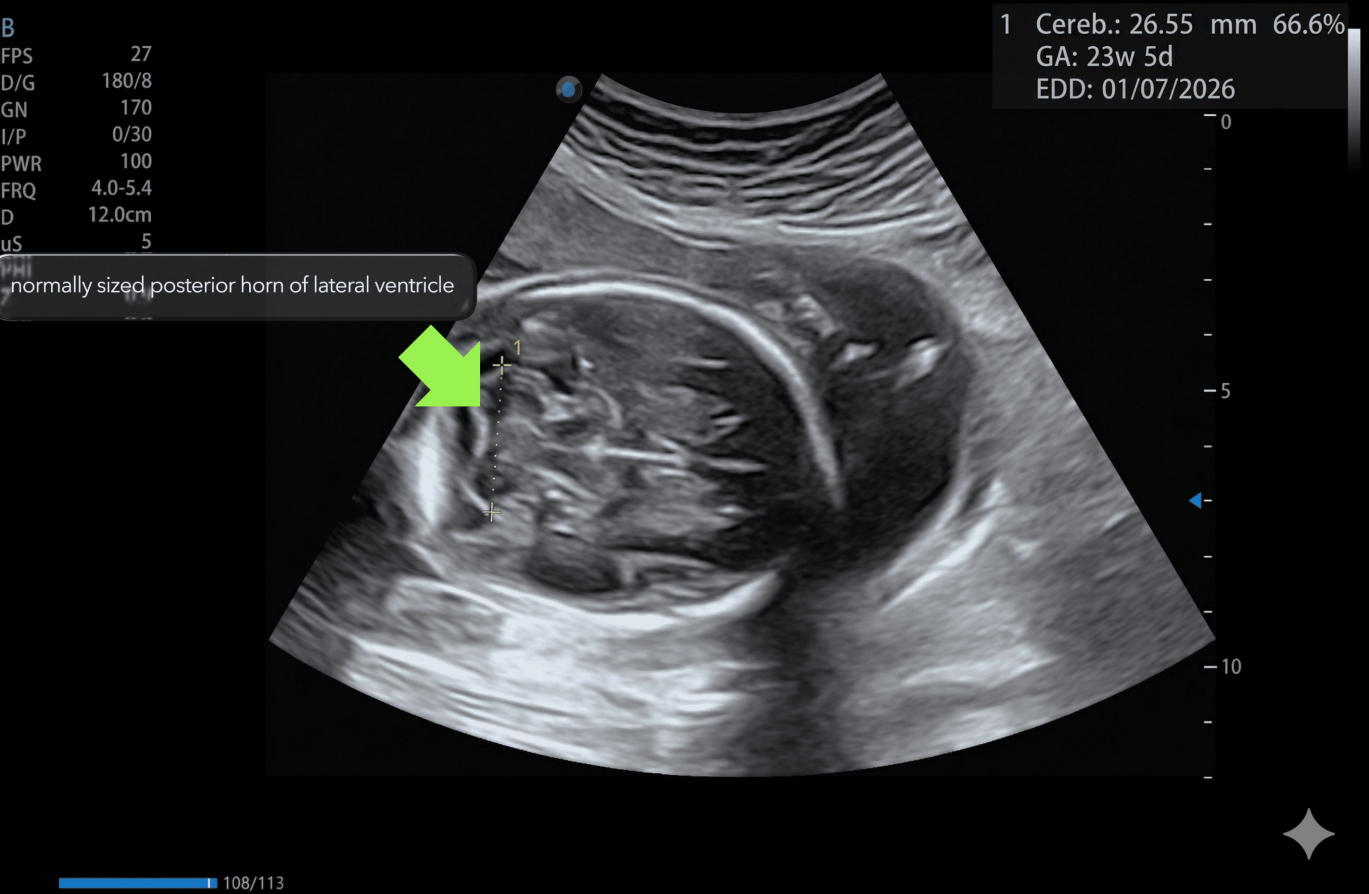
Normal anomaly scan in 23 weeks foetus showing TCD and normal-sized posterior horn of lateral ventricle TCD: transcerebellar diameter

Her current pregnancy had been uneventful up to that point. A detailed second-trimester anomaly scan performed at 23 weeks of gestation demonstrated normal foetal anatomy, appropriate growth parameters, and a normally situated placenta with no evidence of placenta previa or retroplacental hematoma (Figure 1).

Approximately three weeks later, the patient experienced an abrupt deterioration in her condition, characterized by dizziness, generalized weakness, and signs of circulatory collapse. There was no history of trauma, abdominal injury, or vaginal bleeding. On presentation, she was hypotensive and clinically unstable. The absence of external haemorrhage, coupled with progressive hemodynamic compromise, raised immediate concern for concealed intra-abdominal bleeding.

Given the severity of her presentation, a multidisciplinary assessment involving both obstetrics and general surgery teams was undertaken without delay. In view of the strong suspicion of internal haemorrhage and the patient’s deteriorating condition, a decision was made to proceed with urgent laparoscopic exploration while simultaneously initiating aggressive resuscitative measures, including fluid replacement and blood transfusion.

**Figure 2:**
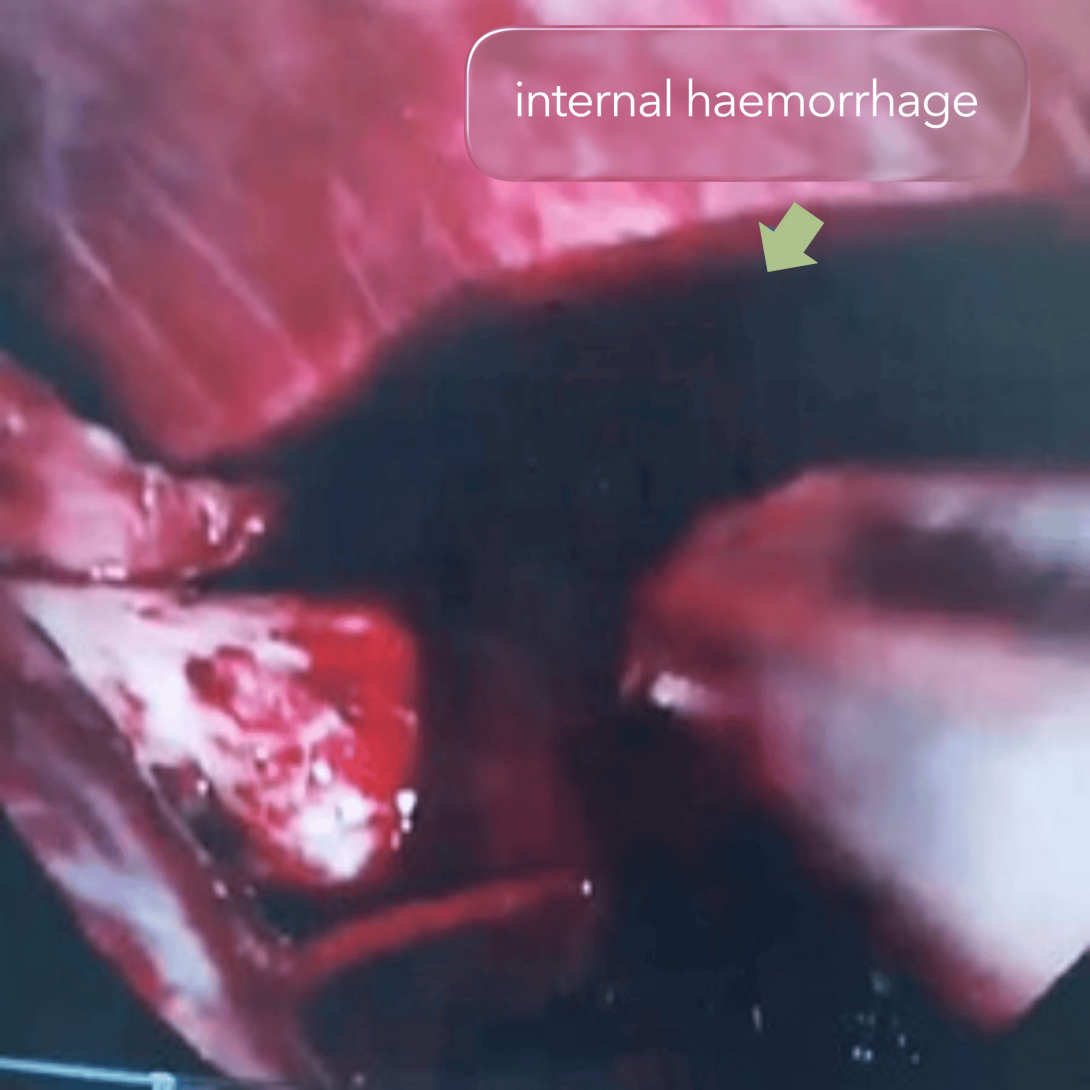
Internal haemorrhage demonstrated during laparoscopy with evident bleeding from the left uterine artery

Intraoperatively, a massive hemoperitoneum was encountered, with an estimated blood loss of approximately 3L. Careful inspection identified an actively bleeding ruptured branch of the left uterine artery. No underlying structural abnormality, vascular malformation, or endometriotic lesion was identified to explain the rupture. Haemostasis was achieved through surgical ligation of the bleeding vessel, followed by thorough evacuation of intraperitoneal blood and irrigation of the abdominal cavity (Figure 2).

The patient received a total of eight units of packed red blood cells during resuscitation. Postoperatively, she was admitted to the intensive care unit for close monitoring and hemodynamic support, where she remained for five days before achieving full stabilization and subsequent transfer to routine care.

Following recovery from the acute event, close foetal surveillance was instituted. Notably, serial foetal Doppler studies remained within normal limits, with no evidence of compromised uteroplacental circulation.

**Figure 3:**
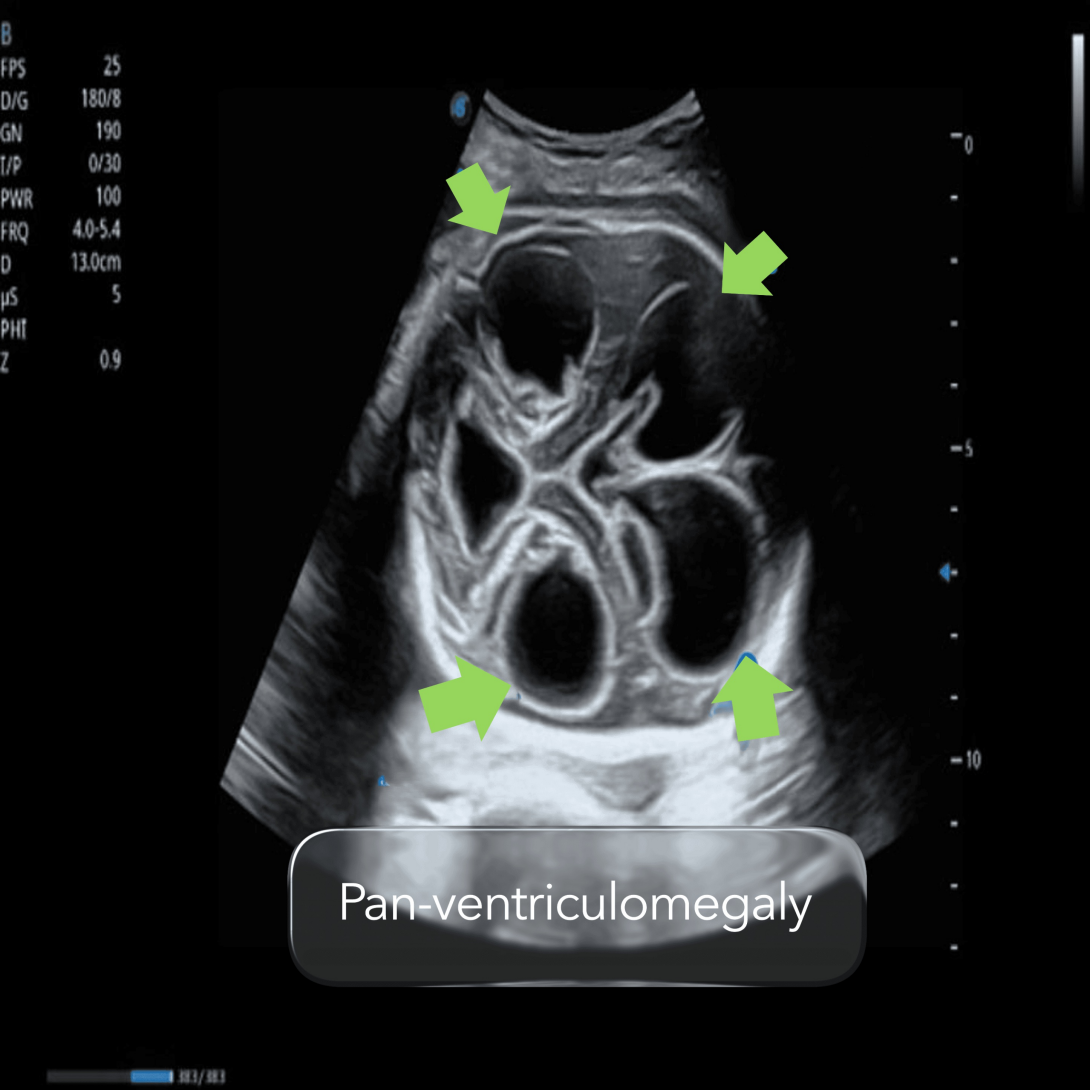
Pan-ventriculomegaly shown in follow-up ultrasound at 32 weeks

However, ultrasound examination at 32 weeks of gestation revealed significant and unexpected foetal intracranial abnormalities. There was marked tetra-ventriculomegaly, with both lateral ventricles measuring approximately 33 mm. The ventricular walls appeared thickened and echogenic, and intraventricular echogenic material consistent with haemorrhagic clots was observed. Additional findings included echogenic changes suggestive of cortical oedema and involvement of the choroid plexus, raising a strong suspicion of intrauterine intracranial haemorrhage (Figure 3). The cerebellum could not be adequately visualized, likely due to compression from the markedly dilated ventricular system.

A follow-up ultrasound performed at 35 weeks confirmed persistence of these findings, with no interval improvement. In addition, there was evidence of evolving foetal growth restriction, as the abdominal circumference declined from the 50th percentile to below the 3rd percentile.

The patient subsequently delivered at 38 weeks of gestation. The neonate had a birth weight of 2.5 kg and an Apgar score of 8 at birth. The newborn was admitted to the neonatal intensive care unit for observation for three days, during which time feeding was well established with no immediate complications. Neurological assessment was reassuring in the early neonatal period.

**Figure 4:**
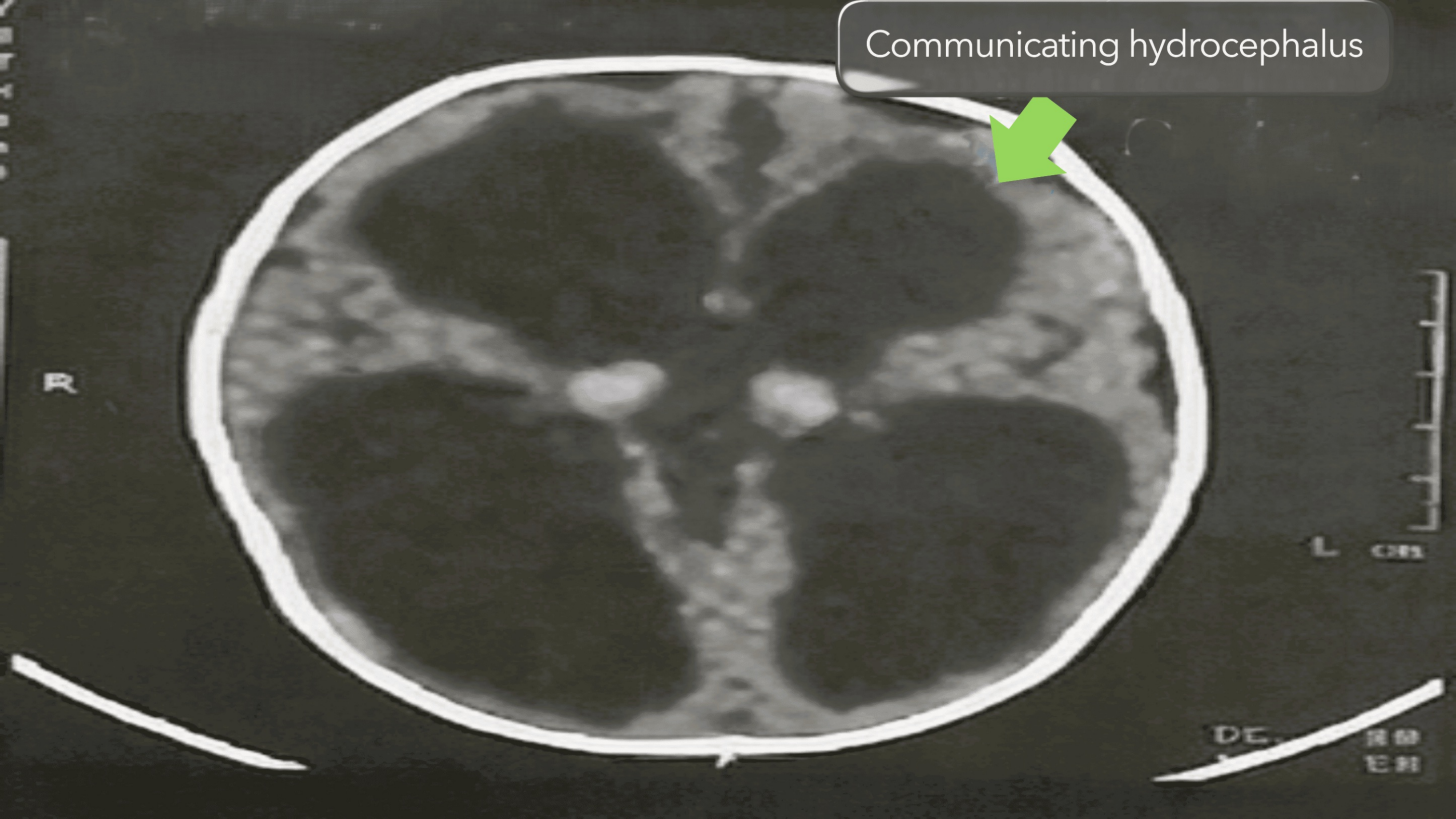
Postnatal CT brain showing communicating hydrocephalus

Postnatal brain imaging, however, confirmed the presence of significant hydrocephalus, consistent with the antenatal findings. As a result, the infant underwent ventriculoperitoneal shunt placement two weeks after delivery (Figure 4). The postoperative course was uneventful, and the infant demonstrated satisfactory clinical progress.

## Discussion

Spontaneous hemoperitoneum in pregnancy (SHiP) is a rare but potentially life-threatening condition characterized by the sudden accumulation of blood within the peritoneal cavity, often in the absence of trauma or identifiable precipitating factors [2]. Among its causes, rupture of uterine or utero-ovarian vessels represents an uncommon but well-documented aetiology [3,4].

In most reported cases, predisposing factors such as endometriosis, vascular anomalies, or prior pelvic surgery are identified [1,2]. However, in a subset of patients, no clear underlying cause can be established, as demonstrated in the present case, where intraoperative findings revealed rupture of a branch of the left uterine artery without any identifiable pathology. This highlights the unpredictable nature of the condition and the importance of maintaining a high index of suspicion.

A particularly notable aspect of this case is the development of severe foetal intracranial pathology despite successful maternal stabilization and persistently normal foetal Doppler studies. This observation underscores an important clinical concept: normal Doppler findings do not exclude prior episodes of acute foetal hypoxia.

The most plausible mechanism involves transient but profound maternal hypovolemia leading to uteroplacental insufficiency and foetal hypoxic-ischemic injury. Such hypoxic events may compromise the integrity of the fragile foetal cerebral vasculature, predisposing to intraventricular haemorrhage. This is consistent with previous reports demonstrating that foetal intracranial haemorrhage can occur secondary to hypoxic or hemodynamic disturbances [5,6].

The ultrasound findings in this case - marked tetraventriculomegaly, echogenic ventricular lining, and intraventricular clots - are characteristic of high-grade foetal intracranial haemorrhage. The persistence of these findings and the subsequent development of foetal growth restriction further support the presence of ongoing or previous placental insufficiency [7,8].

Clinically, SHiP often presents with nonspecific features, including acute abdominal pain and hemodynamic instability without external bleeding. This may lead to delays in diagnosis, as more common obstetric causes such as placental abruption or uterine rupture are initially considered [9]. In this case, the absence of vaginal bleeding, combined with rapid hemodynamic deterioration, appropriately prompted suspicion of concealed intra-abdominal haemorrhage and expedited surgical intervention, which was critical for maternal survival.

Importantly, this case highlights the potential dissociation between Doppler findings and actual foetal neurological status. While Doppler studies are valuable in assessing current foetal haemodynamics, they may not reflect prior transient hypoxic insults, which can still result in significant neurological injury.

From a clinical perspective, this case emphasizes the need for close and continued foetal surveillance following any episode of significant maternal hemodynamic compromise. Even in the presence of reassuring Doppler findings, detailed neuro-sonographic evaluation should be considered to detect early signs of foetal brain injury.

## Conclusions

This case highlights a rare presentation of spontaneous uterine artery rupture resulting in massive intra-abdominal haemorrhage and maternal shock, followed by severe foetal intracranial haemorrhage and ventriculomegaly. It emphasizes early suspicion of internal haemorrhage, the importance of rapid surgical intervention, and the need for detailed foetal neurological follow-up.

## References

[1] Brosens IA, Fusi L, Brosens JJ (2009). Endometriosis is a risk factor for spontaneous hemoperitoneum during pregnancy. Fertil Steril.

[2] Lier MC, Malik RF, Ket JC, Lambalk CB, Brosens IA, Mijatovic V (2017). Spontaneous hemoperitoneum in pregnancy (SHiP) and endometriosis - a systematic review of the recent literature. Eur J Obstet Gynecol Reprod Biol.

[3] Hodgkinson CP, Christensen RC (1950). Hemoperitoneum in pregnancy. Am J Obstet Gynecol.

[4] da Silva CM, Luz R, Almeida M, Pedro D, Paredes B, Branco R, Pereira A (2020). Hemoperitoneum during pregnancy: a rare case of spontaneous rupture of the uterine artery. Case Rep Obstet Gynecol.

[5] Ghi T, Simonazzi G, Perolo A (2003). Outcome of antenatally diagnosed intracranial hemorrhage: case series and review of the literature. Ultrasound Obstet Gynecol.

[6] Kutuk MS, Yikilmaz A, Ozgun MT (2014). Prenatal diagnosis and postnatal outcome of fetal intracranial hemorrhage. Childs Nerv Syst.

[7] Vergani P, Locatelli A, Strobelt N, Cavallone M, Ceruti P, Paterlini G, Ghidini A (1998). Clinical outcome of mild fetal ventriculomegaly. Am J Obstet Gynecol.

[8] Melchiorre K, Bhide A, Gika AD, Pilu G, Papageorghiou AT (2009). Counseling in isolated mild fetal ventriculomegaly. Ultrasound Obstet Gynecol.

[9] Oyelese Y, Ananth CV (2006). Placental abruption. Obstet Gynecol.

